# *Clostridioides difficile* co-infection in patients with COVID-19

**DOI:** 10.2217/fmb-2021-0145

**Published:** 2022-04-20

**Authors:** Roman Maslennikov, Vladimir Ivashkin, Anna Ufimtseva, Elena Poluektova, Anatoly Ulyanin

**Affiliations:** ^1^Department of Internal Medicine, Gastroenterology and Hepatology, Sechenov University, 8-2 Trubetskaya str., Moscow, 119991, Russian Federation; ^2^The Interregional Public Organization ‘Scientific Community for the Promotion of the Clinical Study of the Human Microbiome’, Pogodinskaya Street, 1, Building 1, Moscow, 119435, Russian Federation

**Keywords:** antibiotic-associated diarrhea, *Clostridioides difficile*, *Clostridium difficile*, coronavirus, COVID-19, SARS-CoV-2

## Abstract

**Aim:** To assess the impact of *Clostridioides difficile* infection on the course of COVID-19. **Methods:** The authors included 809 patients with COVID-19 in this retrospective study: 55 had *C. difficile* infection, 23 had *C. difficile*-negative antibiotic-associated diarrhea and 731 had no diarrhea. *C. difficile* in feces was determined by immunochromatographic test for its toxins. **Results:** *C. difficile* infection was associated with increased risk of death (hazard ratio = 2.6; p = 0.021), especially after 20 days of disease (hazard ratio = 6.5; p < 0.001). *C. difficile* infection-associated diarrhea was longer and more severe than *C. difficile*-negative antibiotic-associated diarrhea. Unlike patients with *C. difficile*-negative antibiotic-associated diarrhea, patients with *C. difficile* infection were admitted to the intensive care unit and needed mechanical ventilation more often than those without diarrhea. **Conclusion:** *C. difficile* infection worsens the course and prognosis of COVID-19.

## Plain language summary

Patients with COVID-19 usually receive antibiotic treatment, which predisposes them to antibiotic-associated diarrhea. In some cases, antibiotic-associated diarrhea can be caused by *Clostridioides difficile* bacteria. To learn more about the impact of *C. difficile* infection on COVID-19, the authors analyzed data from the medical records of 809 patients with COVID-19. The authors found that *C. difficile* co-infection worsens the course and prognosis of COVID-19. The authors suggest that patients with COVID-19 who develop diarrhea after taking antibiotics be tested for *C. difficile* and treated for this co-infection if the test is positive.

## Tweetable abstract

*Clostridioides difficile* co-infection worsens the course and prognosis of COVID-19.

## Graphical abstract

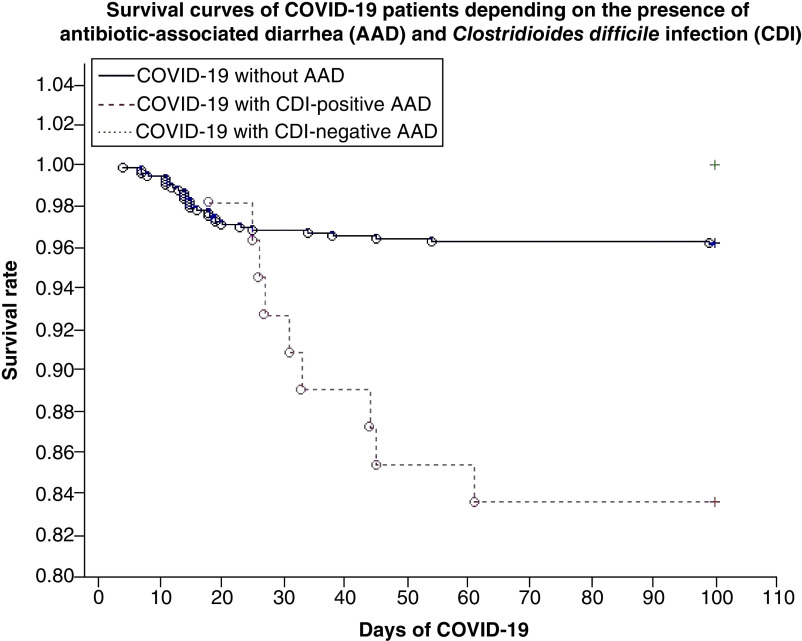

Novel COVID-19 is the first pandemic of the 21st century [1]. Diarrhea occurs in approximately 10% of patients with COVID-19 [2], and it is the first manifestation of the disease in approximately 1% of cases [3,4]. Patients with COVID-19 usually receive massive antibiotic treatment, which predisposes them to antibiotic-associated diarrhea (AAD), including diarrhea caused by *Clostridioides difficile* [5,6]. The first report was limited to the description of nine cases of *C. difficile* infection in COVID-19 without comparison with the group of COVID-19 patients without *C. difficile* infection [7]. A recent study showed that *C. difficile* infection develops in about 10% of patients with COVID-19 and identified risk factors for its development [8]. However, the researchers did not indicate how the infection affects the course of COVID-19, and there was no comparison with *C. difficile*-negative AAD. Italian researchers found *C. difficile* infection in only 0.4% of COVID-19 patients, described risk factors for the development of the co-infection and discussed the fact that its presence leads to an increase in hospitalization time [9]. However, the effect of the infection on mortality in COVID-19 was not described. American researchers reported a higher mortality rate in COVID-19 patients with *C. difficile* infection compared with COVID-19 patients without *C. difficile* infection, but only five such patients were analyzed in this study [10].

Thus, the course of the combination of these deadly infections remains poorly explored. This study aimed to assess the impact of *C. difficile* infection on the course and prognosis of COVID-19 in a large cohort of patients. The authors believe that this work will be important to all doctors fighting COVID-19 around the world.

## Methods

### Patients

This was a retrospective single-center study that included patients admitted to the Clinic of Internal Diseases, Gastroenterology and Hepatology of Sechenov University from April to July 2020 with COVID-19 diagnosed through clinical or laboratory results [11]. All included patients received antibiotics, and some received glucocorticoids, with a minimum course of 5 days for these drugs.

The authors used data from the electronic medical records of patients admitted to the clinic. The authors excluded patients with diarrhea due to other causes as well as those who had viral diarrhea, defined as self-limiting, short-term, mild diarrhea that developed before taking antibiotics or in the first days of taking them (no later than 10 days from the onset of the disease) [3,12]; took laxatives; died during the first day of hospitalization of causes not associated with COVID-19 and diarrhea; and had diarrhea but had not been tested for *C. difficile* toxins in feces. The following diseases were considered chronic: hypertension, coronary artery disease, asthma, chronic obstructive pulmonary disease, chronic hepatitis, cirrhosis, leukemia, chronic anemia, rheumatoid arthritis and other rheumatic diseases, cancer, diabetes, chronic glomerulopathies and other diseases that lasted at least 6 months.

### Ethical approval

The study was approved by the local ethics committee. The study was carried out in accordance with the Declaration of Helsinki (as revised in Brazil in 2013). All patients signed informed consent authorizing the use of their medical data for scientific purposes.

### Patient grouping

The authors considered diarrhea to be the presence of loose or watery stools or an increase in the frequency of bowel movements (more than three per day). COVID-19 patients whose diarrhea was considered AAD [12] and had lasted at least 5 days or occurred at least five times per day were tested for *C. difficile* toxins with a fecal immunochromatographic rapid test (VedaLab, Cerisé, France). The presence of *C. difficile* infection was confirmed by the detection of at least one of its toxins [13]. Patients with AAD who were not tested for *C. difficile* infection were considered patients with unknown AAD and were excluded from the study.

Patients were not tested for other infectious agents that could cause diarrhea because the risk of infection in the authors' hospital was negligible as a result of the high level of epidemic surveillance. Thus, the authors divided the patients included in the study into three groups: those with AAD and a positive test for *C. difficile* infection (group POS), those with AAD and a negative test for *C. difficile* infection (group NEG) and those without diarrhea (group NO).

### Outcomes

The authors assessed mortality as the primary outcome and frequency of admission to the intensive care unit, need for mechanical ventilation and duration of hospitalization as secondary outcomes.

### Statistics

Results are presented as median and interquartile range. Groups were compared using Mann–Whitney *U* test for continuous data and χ2 test for categorical data. The Kaplan–Meier estimator and Cox F test were used for survival estimates. The influence of factors on patient survival and hazard ratio were assessed with the Cox regression model. The criterion for significance was p <  0.050. Statistica 10 (TIBCO Software, CA, USA) and SPSS Statistics 23 (IBM Corporation, NY, USA) were used for statistical calculations.

## Results

**Figure 1.**
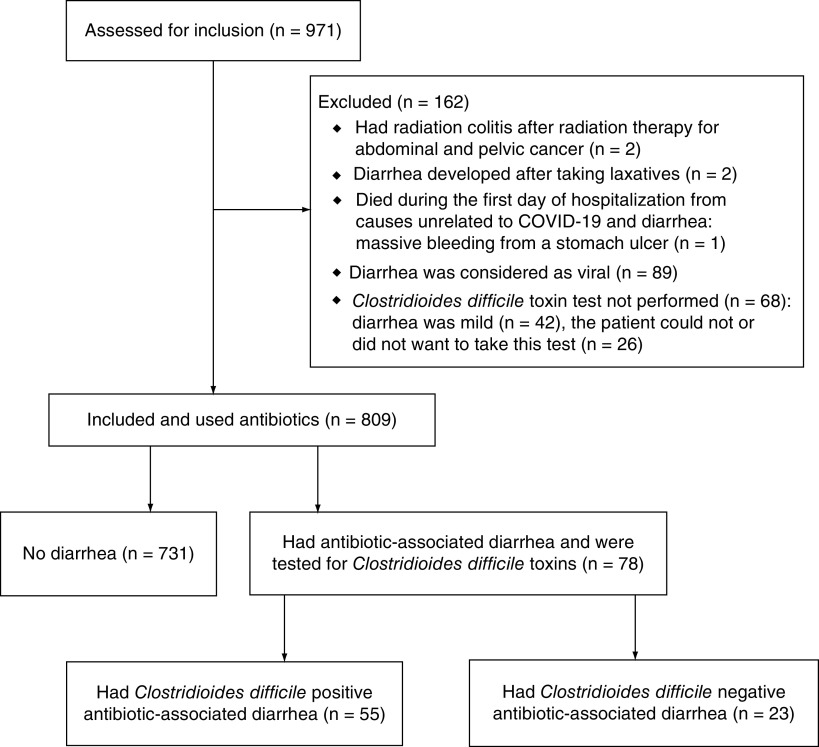
Consolidated Standards of Reporting Trials 2010 flow diagram.

The study included 809 patients with COVID-19 (Figure 1). A total of 55 patients had a positive test for *C. difficile* infection (group POS), 23 patients had a negative test for *C. difficile* infection (group NEG) and 731 patients were without diarrhea (group NO).

The main characteristics of the included patients are shown in Table 1. Unlike those with a negative test, AAD patients with a positive test for *C. difficile* infection were older than those without diarrhea. The proportion of women was higher among COVID-19 patients with *C. difficile* infection than among COVID-19 patients without diarrhea (Table 1).

**Table 1.** Main characteristics of COVID-19 patients.

Characteristics	POS group (n = 55)	NEG group (n = 23)	NO group (n = 731)	POS versus NEG p-value	POS versus NO p-value	NEG versus NO p-value
Age, years (interquartile range)	65 (59–73)	60 (51–70)	54 (44–64)	0.176	<0.001	0.057
Male/female, n	18/37	12/11	363/368	0.108	0.015	0.812
Body temperature on admission, °C (interquartile range)	38 (37.1–38.4)	37.5 (36.8–38.0)	37.5 (37.0–38.0)	0.194	0.005	0.744
BMI, kg/m^2^ (interquartile range)	30.0 (26.1–33.6)	32.3 (29.2–34.4)	28.4 (25.8–32.3)	0.381	0.444	0.118
Duration of disease before admission, days (interquartile range)	7 (4–10)	7 (5–12)	7 (5–10)	0.218	0.683	0.256
Length of hospital stay, days (interquartile range)	20 (16–27)	19 (13–22)	15 (13–18)	0.089	<0.001	0.048
Total duration of disease, days (interquartile range)	31 (23–35)	28 (24–37)	23 (20–28)	0.730	<0.001	0.001
Day of onset of diarrhea from onset of disease (interquartile range)	17 (12–24)	16 (13–22)	–	0.300	–	–
Day of onset of diarrhea from day of hospitalization (interquartile range)	9 (2–14)	6 (2–10)	–	0.771	–	–
Day of onset of diarrhea from day of initiation of antibiotics (interquartile range)	14 (9–19)	13 (8–19)	–	0.417	–	–
Day of initiation of antibiotics from onset of disease (interquartile range)	3 (1–5)	3 (1–7)	4 (2–7)	0.321	0.001	0.333
Duration of antibiotic use before hospitalization, days (interquartile range)	4 (0–8)	4 (0–10)	2 (0–5)	0.532	0.045	0.023
Patients taking antibiotics before hospitalization, n (%)	33 (60.0)	17 (73.9)	397 (54.3)	0.243	0.414	0.063
Patients with hospitalizations in previous 3 months, n (%)	11 (20.0)	2 (8.7)	36 (4.9)	0.222	<0.001	0.416
Duration of diarrhea, days (interquartile range)	8 (6–15)	5 (4–8)	–	0.002	–	–
Bowel movements per day, n (interquartile range)	6 (4–9)	4 (3–7)	–	0.041	–	–
Patients with comorbidities, n (%)	44 (80.0)	20 (87.0)	421 (57.6)	0.466	0.011	0.005
Patients with lung involvement by chest CT scan, n (%)	55 (100)	21 (91.3)	713 (97.6)	0.153	0.478	0.241
Deaths, n (%)	9 (16.4)	0 (0.0)	28 (3.8)	0.044	<0.001	0.343
Deaths within first 20 days of disease, n (%)	1 (1.8)	0 (0.0)	21 (2.9)	–	0.656	0.412
Deaths after 20 days of disease, n (%)	8 (14.5)	0 (0.0)	7 (1.0)	0.056	<0.001	0.633
Patients admitted to ICU, n (%)	15 (27.2)	2 (8.7)	44 (6.0)	0.131	<0.001	0.932
Patients admitted to ICU before onset of diarrhea, n (%)	7 (12.7)	2 (8.7)	44 (6.0)	0.905	0.052	0.932
Day of ICU admission from onset of disease (interquartile range)	13 (7–24)	8 (7–9)	9 (7–12)	0.300	0.029	0.720
Day of ICU admission from first hospitalization day (interquartile range)	4 (0–10)	3.5 (3–4)	2 (0–5)	0.940	0.356	0.620
Days spent in ICU (interquartile range)	6 (2–24)	15 (12–18)	5 (4–12)	0.502	0.625	0.083
Patients needing mechanical ventilation, n (%)	9 (16.4)	0 (0.0)	24 (3.3)	0.039	<0.001	0.475
Patients with colitis, n (%)	21 (38.2)	6 (26.1)	0 (0.0)	0.306	<0.001	<0.001
Patients with paralytic ileus, n (%)	5 (9.1)	0 (0.0)	0 (0.0)	0.323	<0.001	1.000

POS group = COVID-19 patients with *Clostridioides difficile*-positive AAD. NEG group = COVID-19 patients with *C. difficile*-negative AAD. NO group = COVID-19 patients without diarrhea.

AAD: Antibiotic-associated diarrhea; ICU: Intensive care unit.

### Mortality

**Figure 2.**
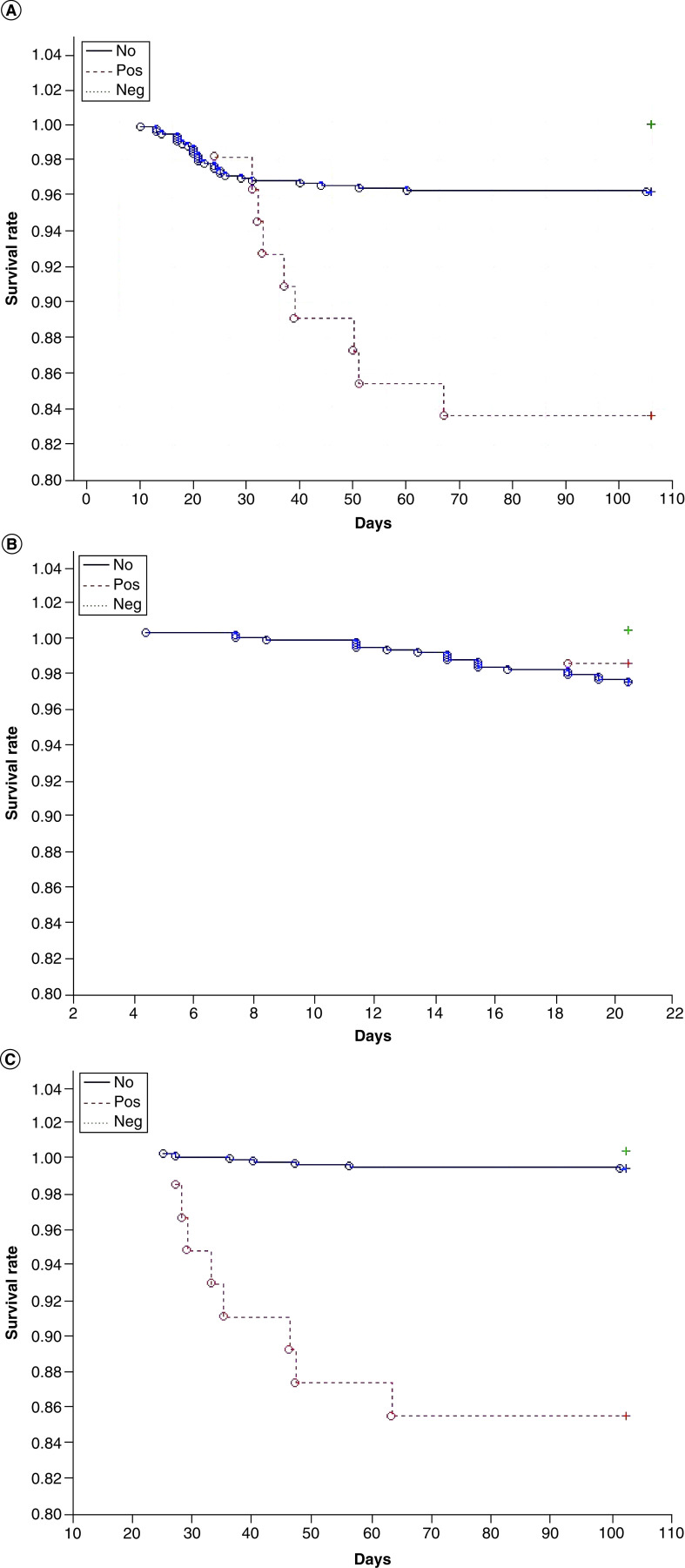
Deaths of COVID-19 patients with *Clostridioides difficile*-positive AAD (Pos group), COVID-19 patients with *C. difficile*-negative AAD (Neg group) and COVID-19 patients without diarrhea (no group). **(A)** Total deaths. **(B)** Deaths occurring during first 20 days of COVID-19 infection. **(C)** Deaths occurring after 20th day of COVID-19 infection. AAD: Antibiotic-associated diarrhea.

A total of 37 (4.5%) patients died, including nine (16.4%) with *C. difficile* infection and 28 (3.8%) without diarrhea. There were no deaths in the NEG group, possibly because of its small size. The overall mortality rate in patients with *C. difficile* infection was higher than that observed in patients without diarrhea and those with *C. difficile*-negative AAD. There was no significant difference between the groups with regard to the proportion of patients who died in the first 20 days of COVID-19 infection. However, among patients who died after the 20th day of illness, those with *C. difficile* infection accounted for more than half (Figure 2 & Table 1).

Patients with *C. difficile* infection died later than patients without diarrhea (median: 31 vs 15 days; interquartile range: 26–44 vs 12–22 days; p < 0.001). The main factors that increased mortality in the general cohort of patients were older age (p < 0.001), *C. difficile* infection (hazard ratio = 2.6; p = 0.021) and high CRP level on admission (p = 0.003).

*C. difficile* infection (hazard ratio = 6.5; p < 0.001), longer length of hospitalization (p = 0.001) and older age (p = 0.002) were independent risk factors for death after 20 days of disease. Age (p < 0.001) and high CRP on admission (p < 0.001), but not *C. difficile* infection (p = 0.262), were the main predictors of death before the 21st day of disease. Glucocorticoid use was not a predictor of death before or after 20 days of disease (p = 0.163 and 0.266, respectively).

Four of nine deceased patients with *C. difficile* infection had diarrhea at the time of death. Diarrhea was replaced by paralytic ileus 1–5 days before death in the remaining five patients. Seven of nine deceased patients with *C. difficile* infection had colitis detected.

### Course of disease

Patients with AAD had a longer hospital stay and total disease duration, were administered antibiotics longer before admission and had comorbidities more often than patients without diarrhea. There was no significant difference in the values of these indicators between *C. difficile*-positive and negative AAD patients. Unlike those with a negative test, AAD patients with a positive test for *C. difficile* infection had higher body temperature on admission, started taking antibiotics earlier, were hospitalized more frequently prior to the current hospitalization, were admitted to the intensive care unit more often and needed mechanical ventilation more often than patients without diarrhea (Table 1). Abdominal pain was more frequent in the POS group than the NO group. There was no difference between groups in the incidence of other COVID-19 symptoms (Table 2).

**Table 2.** Symptoms and comorbidities in COVID-19 patients.

Symptoms/comorbidities, n (%)	POS group (n = 55)	NEG group (n = 23)	NO group (n = 731)	POS versus NEG p-value	POS versus NO p-value	NEG versus NO p-value
Fever	53 (96.4)	22 (95.7)	675 (92.3)	0.655	0.405	0.848
Cough	37 (67.3)	14 (60.9)	489 (66.9)	0.588	0.954	0.546
Runny nose	2 (3.6)	1 (4.3)	69 (9.3)	0.655	0.229	0.643
Sore throat	2 (3.6)	1 (4.3)	91 (12.4)	0.655	0.083	0.399
Chest pain	8 (14.5)	1 (4.3)	91 (12.4)	0.370	0.651	0.398
Dyspnea	27 (49.1)	11 (47.8)	377 (51.6)	0.919	0.722	0.723
Headache	7 (12.7)	2 (8.7)	177 (24.2)	0.905	0.052	0.141
Anosmia	3 (5.5)	3 (13.0)	96 (13.1)	0.496	0.149	0.990
Ageusia	2 (3.6)	0 (0.0)	45 (6.2)	0.888	0.642	0.435
Anorexia	1 (1.8)	0 (0.0)	24 (3.3)	0.651	0.843	0.780
Myalgia	3 (5.5)	0 (0.0)	102 (14.0)	0.620	0.114	0.106
Arthralgia	2 (3.6)	0 (0.0)	48 (6.6)	0.888	0.567	0.403
Abdominal pain	5 (9.1)	0 (0.0)	13 (1.8)	0.323	0.003	0.886
Vomiting	2 (3.6)	0 (0.0)	42 (5.7)	0.888	0.725	0.471
Cardiovascular disease	32 (58.2)	15 (65.2)	329 (45.0)	0.563	0.059	0.055
Respiratory disease	3 (5.5)	0 (0.0)	40 (5.5)	0.620	0.996	0.249
Liver disease	2 (3.6)	2 (8.7)	2 (0.3)	0.718	0.017	0.001
Kidney disease	6 (10.9)	0 (0.0)	39 (5.3)	0.237	0.086	0.510
Rheumatic disease	0 (0.0)	0 (0.0)	24 (3.3)	–	0.338	0.780
Blood disease	1 (1.8)	0 (0.0)	9 (1.2)	0.651	0.803	0.592
Cancer	3 (5.5)	0 (0.0)	40 (5.5)	0.620	0.058	0.660
Diabetes mellitus	6 (10.9)	1 (4.3)	91 (12.4)	0.624	0.738	0.398

POS group = COVID-19 patients with *Clostridioides difficile*-positive AAD. NEG group = COVID-19 patients with *C. difficile*-negative AAD. NO group = COVID-19 patients without diarrhea.

AAD: Antibiotic-associated diarrhea.

Among chronic comorbidities, liver diseases were more often detected in the POS and NEG groups than in the NO group. There was no significant difference in the incidence of other chronic diseases between patient groups (Table 2).

Serum total protein (p < 0.001) and potassium (p < 0.001) levels were slightly reduced and lactate dehydrogenase activity (p = 0.004) was increased in patients in the POS group compared with patients without diarrhea. There was no significant difference in the values of other laboratory parameters between the patient groups (Table 3).

**Table 3.** Laboratory data of COVID-19 patients.

Characteristics	POS group (n = 55)	NEG group (n = 23)	NO group (n = 731)	p-value
Creatinine, μmol/l (interquartile range)	99 (86–114)	98 (89–112)	93 (82–107)	0.042
Total protein, g/l (interquartile range)	68 (63–71)	70 (65–73)	71 (67–75)	0.001
Albumin, g/l (interquartile range)	41 (37–44)	42 (40–44)	41 (39–44)	0.290
Total bilirubin, μmol/l (interquartile range)	9 (8–13)	12 (9–13)	9 (7–12)	0.086
Alanine aminotransferase, U/l (interquartile range)	34 (25–46)	31 (25–73)	34 (23–49)	0.893
Asparanine aminotransferase, U/l (interquartile range)	38 (28–49)	31 (28–57)	35 (27–47)	0.846
Creatine kinase, U/l (interquartile range)	198 (93–318)	124 (96–329)	102 (68–196)	0.179
Lactate dehydrogenase, U/l (interquartile range)	564 (452–713)	462 (339–507)	442 (368–549)	0.013
Amylase, U/l (interquartile range)	43 (30–72)	49 (25–53)	51 (37–60)	0.673
Glucose, mmol/l (interquartile range)	5.9 (5.2–7.3)	5.5 (5.0–6.1)	5.5 (4.8–6.4)	0.067
Sodium, mmol/l (interquartile range)	140 (138–143)	141 (137–142)	141 (138–145)	0.194
Potassium, mmol/l (interquartile range)	4.0 (3.8–4.4)	4.2 (4.0–4.6)	4.4 (4.1–4.9)	< 0.001
Iron, μmol/l (interquartile range)	3.4 (1.6–5.8)	4.6 (4.3–9.3)	4.4 (2.3–9.0)	0.106

POS group = COVID-19 patients with *Clostridioides difficile*-positive AAD. NEG group = COVID-19 patients with *C. difficile*-negative AAD. NO group = COVID-19 patients without diarrhea.

AAD: Antibiotic-associated diarrhea.

There was no difference in the frequency of detection of the causative agent of COVID-19 in oral and nasopharyngeal swabs with PCR between the POS and NEG groups (74.2 vs 82.6%; p = 0.634), but it was significantly higher in patients with AAD than in the group without diarrhea (76.9 vs 57.9%; p = 0.001). AAD patients with a positive test for *C. difficile* infection had diarrhea longer and more severely than those with a negative test (Table 1).

### Drug use before AAD

All included patients received antibiotics (Table 4). Hydroxychloroquine was received by 66.6% of patients. Other antiviral drugs were administered in less than 1% of patients. Logistic regression showed that risk factors for the development of *C. difficile* infection in COVID-19 (Table 4) were the use of oral levofloxacin (odds ratio = 1.89), oral amoxicillin/clavulanate (odds ratio = 1.89) and glucocorticoids (odds ratio = 3.4).

**Table 4.** Drugs administered to patients before the onset of *Clostridioides difficile* infection and to patients without diarrhea.

Drugs	*C. difficile* infection (n = 55)	No diarrhea (n = 731)	Rate, %^†^	p-value^‡^
Individual AB				
Azithromycin	42	622	6.3	0.173
Levofloxacin	27	206	11.6	0.018
Levofloxacin, oral	15	117	11.4	0.048
Levofloxacin, par.	16	109	12.8	0.106
Moxifloxacin	10	103	8.8	0.751
Moxifloxacin, oral	7	19	26.9	0.296
Moxifloxacin, par.	3	91	3.2	0.178
Amoxicillin/clavulanate	14	210	6.3	0.940
Amoxicillin/clavulanate, oral	14	136	9.3	0.045
Amoxicillin/clavulanate, par.	3	95	3.1	0.226
Meropenem	9	46	16.4	0.118
Ceftriaxone	38	523	6.8	0.833
GC	37	245	13.1	< 0.001
Clarithromycin	3	72	4.0	0.328
Clarithromycin, oral	2	28	6.7	0.749
Clarithromycin, par.	1	48	2.0	0.427
Josamycin, oral	3	12	20.0	0.233
Cefixime, oral	1	18	5.3	0.947
AB therapy regimens				
Azithromycin + ceftriaxone	1	146	0.7	0.001
Azithromycin + ceftriaxone + GC	4	37	9.8	0.692
Azithromycin + ceftriaxone + other oral AB	4	87	4.4	0.414
Azithromycin + ceftriaxone + GC + other oral AB	5	36	12.2	0.305
Azithromycin + ceftriaxone + other par. AB	2	56	3.4	0.405
Azithromycin + ceftriaxone + other par. AB + GC	6	34	15.0	0.086
Azithromycin + ceftriaxone + other par. AB + other oral AB	1	26	3.7	0.765
Azithromycin + ceftriaxone + other par. AB + other oral AB + GC	4	30	11.8	0.441
Azithromycin + other par. AB	1	40	2.4	0.389
Azithromycin + other par. AB + GC	4	31	11.4	0.476
Azithromycin + other par. AB + other oral AB	1	20	4.8	0.979
Azithromycin + other par. AB + other oral AB + GC	5	21	19.2	0.036
Other combinations of oral and par. ABs ± GCs	6	48	11.1	0.341
Azithromycin only	2	25	7.4	0.765
Other oral AB ± azithromycin	3	36	7.7	0.883
Any oral AB + GC	2	7	22.2	0.253
Other combinations of par. ABs	2	22	8.3	0.884
Other combinations of par. ABs + GCs	2	29	6.5	0.812

† Incidence of diarrhea in patients administered single medication or group of medications.

‡ Significance of difference in the incidence of diarrhea between patients administered and not administered single medication or group of medications.

AB: Antibiotic; GC: Glucocorticoid; par.: Parenteral.

*C. difficile* infection in COVID-19 was least likely to develop if a combination of azithromycin and ceftriaxone was used without glucocorticoids and other antibiotics, whereas it developed significantly more frequently when regimens without ceftriaxone but with glucocorticoids and an oral antibiotic other than azithromycin were used (Table 4).

### AAD treatment

In most of the included cases, a combination of oral antibiotics (metronidazole and/or vancomycin) and the probiotic *Saccharomyces boulardii* were used to treat both *C. difficile*-positive and negative AAD [13,14]. Less commonly, these drugs were used separately. Too many treatment regimens and a small number of included cases made it impossible to conduct a comparative analysis of the effectiveness of different treatment regimens with the required reliability. One patient experienced a recurrence of *C. difficile* infection after successful treatment with oral vancomycin. This relapse was successfully treated with a combination of oral vancomycin and metronidazole [15].

## Discussion

In the authors' study, the development of AAD and *C. difficile* infection complicated the course of COVID-19 in 16.7 and 5.7% of cases, respectively. This is consistent with previously published data [4] and more often than that observed among patients of non-COVID hospitals (0.3 and 0.1%, respectively; p < 0.001) in our country [16]. The development of *C. difficile*-negative AAD did not have a significant effect on the prognosis of COVID-19. However, the risk of death was increased 2.6 times in COVID-19 patients with *C. difficile* infection.

Risk factors for the development of *C. difficile* infection in COVID-19 patients were oral levofloxacin, oral amoxicillin/clavulanate and glucocorticoids. *C. difficile* infection was least likely to develop if a combination of azithromycin and ceftriaxone was used without glucocorticoids and other antibiotics. *C. difficile* infection occurred significantly more frequently when regimens without ceftriaxone but with glucocorticoids and an oral antibiotic other than azithromycin were used. Unfortunately, too many treatment regimens and a small number of observations did not allow the authors to draw a conclusion about which of these is optimal in the treatment of *C. difficile* infection in COVID-19.

The identified risk factors for development of *C. difficile* infection (immunosuppressants, amoxicillin/clavulanate, levofloxacin, hospitalization within previous 3 months) correspond to the published data for the general population [2]. Therefore, the authors recommend testing for *C. difficile* toxins in the feces of all patients with COVID-19 who develop diarrhea during antibiotic therapy.

The limitations of this study are its retrospective nature and the lack of standards for the treatment of *C. difficile* infection in COVID-19. Randomized trials are required to confirm the authors' results and determine the optimal treatment regimen for the combination of these infections. The strengths of the authors' study are its volume and detailed description, which differ significantly from previous studies [3–5]. In addition, the authors have indicated the safest and most risky treatment regimens and have identified a field for future research on this subject.

## Conclusion

*C. difficile* infection worsens the course and prognosis of COVID-19. Further research is needed to develop clinical guidelines for the prevention and treatment of *C. difficile* infection in COVID-19.

**Summary points**

- Patients with antibiotic-associated diarrhea (AAD) had a longer hospital stay and total disease duration than patients without diarrhea. There was no significant difference in the values of these indicators between *Clostridioides difficile*-positive and negative AAD patients.
- Unlike AAD patients with a negative test for *C. difficile* infection, AAD patients with a positive test were admitted to the intensive care unit and needed mechanical ventilation more often than patients without diarrhea.
- AAD patients with a positive test for *C. difficile* infection had diarrhea longer and more severely than those with a negative test.
- The overall mortality rate in patients with *C. difficile* infection was higher than that observed in patients without diarrhea and patients with *C. difficile*-negative AAD. There was no significant difference between the groups with regard to the proportion of patients who died in the first 20 days of COVID-19 infection. However, among patients who died after the 20th day of illness, those with *C. difficile* infection accounted for more than half.
- Patients with *C. difficile* infection died later than patients without diarrhea.
- The main factors that increased mortality were older age, *C. difficile* infection and a high CRP level on admission.
