# Supplementary material for: Clostridioides difficile co-infection in patients with COVID-19
Source: Future Microbiol. 2022 Apr 20:10.2217/fmb-2021-0145. doi: 10.2217/fmb-2021-0145 (PMC9020461; doi:10.2217/fmb-2021-0145)
Supplement: Supplementary file 1 [file fmb-2021-0145ga.pdf]

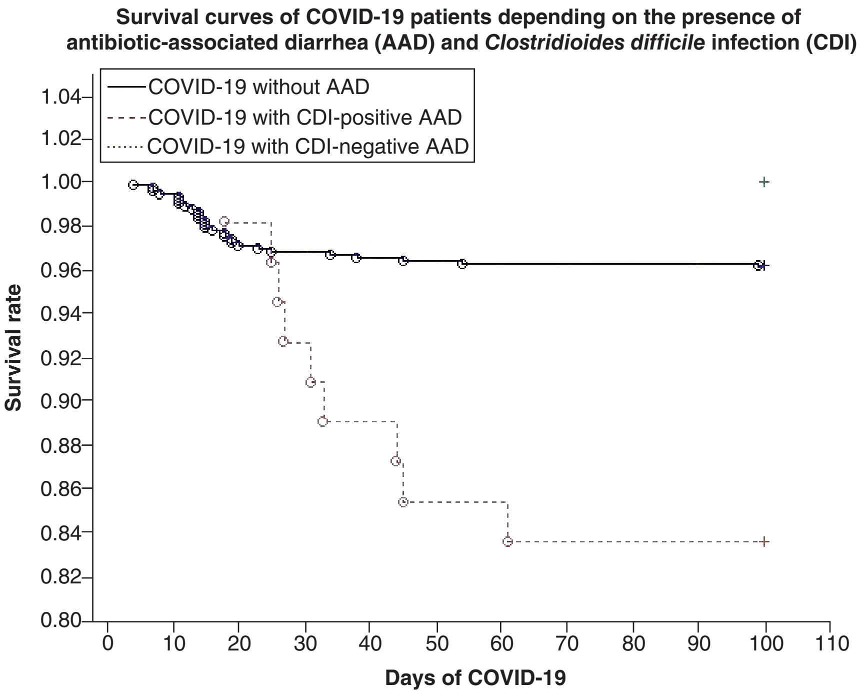
Survival curves of COVID-19 patients depending on the presence of antibiotic-associated diarrhea (AAD) and *Clostridioides difficile* infection (CDI)
COVID-19 without AAD
COVID-19 with CDI-positive AAD
COVID-19 with CDI-negative AAD
Survival rate
1.04
1.02
1.00
0.98
0.96
0.94
0.92
0.90
0.88
0.86
0.84
0.82
0.80
0
10
20
30
40
50
60
70
80
90
100
110
Days of COVID-19
